# Intelligent Fault-Tolerant Control of Delta Robots: A Hybrid Optimization Approach for Enhanced Trajectory Tracking

**DOI:** 10.3390/s25061940

**Published:** 2025-03-20

**Authors:** Carlos Domínguez, Claudio Urrea

**Affiliations:** Electrical Engineering Department, Faculty of Engineering, University of Santiago of Chile (USACH), Las Sophoras 165, Estación Central, Santiago 9170124, Chile; carlos.dominguez@usach.cl

**Keywords:** active fault-tolerant control, delta robot, fault diagnosis, genetic algorithms, gradient descent, hybrid optimization, linear discriminant, meta-learning, principal component analysis, wavelet scattering networks

## Abstract

The kinematic complexity and multi-actuator dependence of Delta-type manipulators render them vulnerable to performance degradation from faults. This study presents a novel approach to Active Fault-Tolerant Control (AFTC) for Delta-type parallel robots, integrating an advanced fault diagnosis system with a robust control strategy. In the first stage, a fault diagnosis system is developed, leveraging a hybrid feature extraction algorithm that combines Wavelet Scattering Networks (WSNs), Principal Component Analysis (PCA), Linear Discriminant Analysis (LDA), and Meta-Learning (ML). This system effectively identifies and classifies faults affecting single actuators, sensors, and multiple components under real-time conditions. The proposed AFTC approach employs a hybrid optimization framework that integrates Genetic Algorithms and Gradient Descent to reconfigure a Type-2 fuzzy controller. Results show that the methodology achieves perfect fault diagnosis accuracy across four classifiers and enhances robot performance by reducing critical degradation to moderate levels under multiple faults. These findings validate the robustness and efficiency of the proposed fault-tolerant control strategy, highlighting its potential for enhancing trajectory tracking accuracy in complex robotic systems under adverse conditions.

## 1. Introduction

Industrial robotics has experienced significant growth in recent decades, driven by the need to automate processes that demand high precision, efficiency, and speed. Within this context, parallel robots, such as the Delta type, have established themselves as essential tools in tasks that require fast and precise manipulations, particularly in industries such as manufacturing, electronics, and food. However, the high dependence on robots in critical processes also implies a challenge: maintaining safe, effective operations despite failures. This need has led to the development of Fault Tolerant Control (FTC) systems, which allow robots to maintain acceptable performance even when failures occur in their components.

Fault tolerance in robotic systems entails detecting, identifying, and compensating for faults to maintain operational continuity with minimal performance loss. FTC mechanisms can be classified into two main categories: passive fault-tolerant control and active fault-tolerant control. In passive fault-tolerant systems, the controller design is conducted considering the possibility of failures, allowing the robot to maintain a certain level of functionality without the need for additional intervention. On the other hand, active fault-tolerant systems use adaptive and reconfigurable strategies that detect and diagnose faults in real time, adjusting the control law to compensate for the detected fault [1,2,3].

The application of fault tolerance techniques is especially relevant in parallel Delta-type robots due to their complex kinematic structure and their reliance on multiple actuators for coordinated motion. Loss of functionality in their actuators can result in significant performance deterioration or, in the worst case, a complete system shutdown. Thus, developing adaptive control mechanisms is essential to sustain robot functionality during failures, minimizing production impacts and costly downtime.

## 2. Literature Review

Numerous studies have investigated fault-tolerant control strategies for industrial robots, with notable approaches leveraging Sliding Mode techniques and extended state observers to enhance robustness against sensor and actuator faults [4,5,6,7,8,9].

The use of adaptive controllers has also been popular among researchers. In [10], an adaptive switching controller is designed through the error transformation method and multiple Lyapunov functions to achieve the system having practical stability within the prescribed time under a class of switching signals satisfying the average residence time.

An adaptive fault-tolerant visual control scheme for robotic manipulators with faulty actuators is proposed in [11]. The authors propose a new algorithm to compensate for jamming faults. The stability of the dynamic system is tested by the Lyapunov method. The effectiveness of the proposed control scheme is verified by comparing and analyzing the manipulator tracking performance under different fault parameters.

In [12], an adaptive robust strategy is explored for a robotic manipulator system in the presence of faults. In the proposed control system, the gains of the Proportional, Integral, Derivative (PID) controller are automatically updated by two adaptive estimators.

On the other hand, the use of artificial intelligence algorithms in fault-tolerant control has been another pillar of recent advances. Methods based on neural networks and deep learning have been used to create prediction models that allow the system to deal with failures before they significantly affect the robot’s performance. These approaches are complemented by techniques such as iterative learning control to improve accuracy and efficiency in error correction.

As an example of these studies that use artificial intelligence, we can mention [13]. The authors of this study propose a robust fuzzy controller to solve the tracking problem of a manipulator with faults in actuators, in the process, and with dynamic uncertainty. An adaptive fuzzy logic system is employed to approximate the unknown dynamics of the robot. A robust adaptive term is designed to compensate for the actuator faults and approximation errors and ensure the convergence and stability of the entire robot control system. According to the authors, the simulation results demonstrate that the proposed robust adaptive fuzzy logic approach for the robotic manipulator has good control performance.

In [14], an adaptive fuzzy system is designed for a second-order Multiple Input-Output Nonlinear System (MIMO-NLS) with unknown initial states, actuator faults, and control saturation. Firstly, a Predefined Time Convergence stability criterion (PTC) is theoretically tested. Then, the authors introduce an error conversion function to convert the trajectory tracking error into a new error variable with an initial value of zero. The numerical simulation results of performance-guaranteed trajectory tracking control for industrial robots with actuator faults demonstrate that the adaptive fault-tolerant fuzzy control algorithm has strong actuator fault tolerance and anti-interference capabilities.

Other researchers on the topic prefer to unify techniques to develop hybrid methods that achieve better results. Such is the case of [15], which is based on a hybrid control scheme that uses an observer as well as a hardware redundancy strategy to improve performance and efficiency in the presence of actuator and sensor failures. Considering a 5 Degrees of Freedom (DoF) robotic manipulator, an adaptive back-stepping methodology is used for fault estimation and the nominal control law for controller reconfiguration. The results affirm the effectiveness of the proposed strategy with model-based friction compensation.

In [16], an extended state observer is constructed to approximate both the velocities and the unknown input to the robotic system. The estimated signals are then combined with a Computed Torque Control (CTC) to construct an active fault-tolerant controller that reduces the influences of the unknown input. Simulations implemented on a 2 DoF manipulator demonstrated that the combination of these techniques offers better trajectory following performance than traditional designs.

### 2.1. Model Predictive Control Approaches

The nonlinear dynamics and actuator coupling of Delta robots challenge trajectory tracking and fault tolerance, motivating the use of Model Predictive Control (MPC) and Nonlinear Model Predictive Control (NMPC). MPC, as reviewed by [17], predicts future states to optimize control inputs under constraints, offering the potential for precise tracking and fault mitigation in Delta robots despite actuator failures or disturbances. MPC could mitigate actuator faults in Delta robots by preemptively adjusting inputs based on predicted behavior [17], yet its real-time application remains limited by computational demands critical for high-speed tasks like pick-and-place.

Several studies have investigated the use of MPC and NMPC for FTC in robotic manipulators, exploring both passive and active fault-tolerance strategies [18,19,20]. NMPC extends MPC by explicitly addressing nonlinear dynamics, making it particularly relevant for Delta robots with their inherent non-linearities and inter-joint dependencies. Ref. [21] proposed a hierarchical framework combining global path planning with NMPC-based tracking for mobile robots, achieving robust navigation in dynamic environments by optimizing trajectories under kinematic constraints.

Applied to Delta robots, NMPC could enhance fault-tolerant control by modeling the complex dynamics of parallel manipulators and optimizing control actions to maintain stability and accuracy despite faults, as suggested by its success in handling unpredictable obstacles [21]. Nevertheless, the literature on NMPC for parallel robots is scarce, and its real-time implementation faces challenges due to the high computational complexity of nonlinear optimization [22,23], especially under the rapid cycle times typical of Delta robot applications.

While these predictive approaches offer theoretical advantages for managing the control difficulties of Delta robots, their practical deployment requires further investigation, particularly in adapting optimization algorithms to the specific dynamics and operational demands of parallel manipulators.

The complexity of the kinematic structure of Delta robots reinforces the need for specialized methodologies to achieve fault tolerance in their components. The works mentioned above were applied to serial manipulators, so those strategies may not be useful for parallel robot cases.

The literature on FTC for Delta robots remains scarce, underscoring a research gap addressed in this study. In one of the studies found [24], a combination of Nonsingular Integral-Type Terminal Sliding Mode (NITSM) and Adaptive high-order Super-Twisting (AST) control is proposed. To obtain better performance, the controller parameters are obtained by Harmony Search Algorithms (HSAs), minimizing an objective function consisting of the Integral Time Absolute Error (ITAE) and the rate of the control signal.

In another study [25], a redundant design is implemented to solve the fault-tolerant problem. The redundant kinematic chain is made according to the structure characteristics, and then the 3-PR(P)S redundant parallel robot is obtained. The motion equations of the control input parameters are deduced under the master control and master-slave control. The simulation results show that the designed parallel redundant structure is reasonable, and the fault-tolerant control of the parallel robot with a 3-PRS structure can be realized.

Considering the gaps in the literature, including the limited exploration of advanced tracking control methods like MPC and NMPC for Delta robots as discussed in Section 2.1, and the critical role of Delta robots in industry, this study proposes an active fault-tolerant control strategy to maintain manipulator functionality under various fault conditions. The main contributions of this paper are as follows:

- A fault diagnostic system is developed using a combination of Wavelet Scattering Networks (WSNs), Principal Component Analysis (PCA), Linear Discriminant Analysis (LDA), and Meta-Learning.
- Four fault classifiers based on bagged trees, linear discriminant, medium neural network, and cosine K-nearest neighbor are studied and compared.
- An active fault-tolerant control strategy based on hybrid optimization, using genetic algorithms and gradient descent, is proposed.
- A cost function for optimization is presented, penalizing tracking error, control effort, and abrupt changes in the controller’s scaling factors.

The rest of this paper is organized as follows: Section 3 describes the studied Delta robot. Section 4 presents the design of the fault diagnostic system. The active fault-tolerant control strategy is described in Section 5. Section 6 discusses the results, and Section 7 presents conclusions and future work.

## 3. Description of the Delta Robot Under Study

The Delta robot, a parallel manipulator, features a mobile platform linked to a fixed base via multiple identical kinematic chains, enabling high-speed and precise motion. Each of these chains is composed of an arm connected to a four-bar mechanism in the form of a parallelogram through a revolution joint. This structural design contributes to the robot’s ability to reach very high speeds and accelerations, surpassing other types of manipulators, making it an ideal tool for applications requiring high precision and speed, such as pick-and-place tasks [26,27].

**Figure 1:**
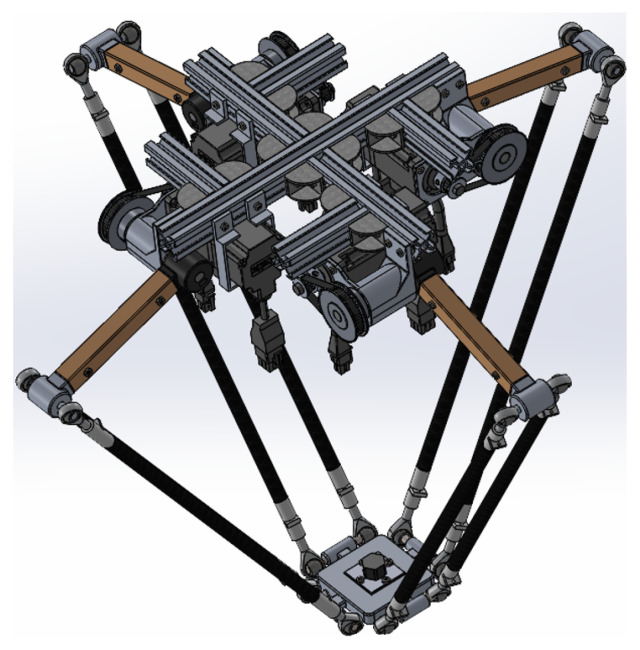
Delta robot designed in SolidWorks.

The design of the manipulator, the result of which can be seen in Figure 1, was carried out in SolidWorks 2024 SP1.0 based on the kinematic and dynamic analysis developed by the authors in previous works. The robot has four arms arranged at 90° of each one and has 3 DoF. The structure of the bases and arms are made of 3003 aluminum, while the forearms are made of carbon fiber. For detailed specifications, modeling, and construction details, refer to [28,29].

Based on the Delta robot design specifications, the BCH2LD0431CA5C servomotor from Schneider Electric, Santiago, Chile has been selected as the actuator due to its ability to provide precise motion control, high speed, and energy efficiency. With a nominal power of 400 W and a maximum speed of 5000 RPM, this servomotor is ideal for ensuring optimal performance in the robot’s dynamics. Additionally, its compact design and compatibility with Lexium LXM28 servo drives from Schneider Electric, Santiago, Chile, enable efficient integration into the control system, ensuring a fast and stable response to the task’s demands.

**Figure 2:**
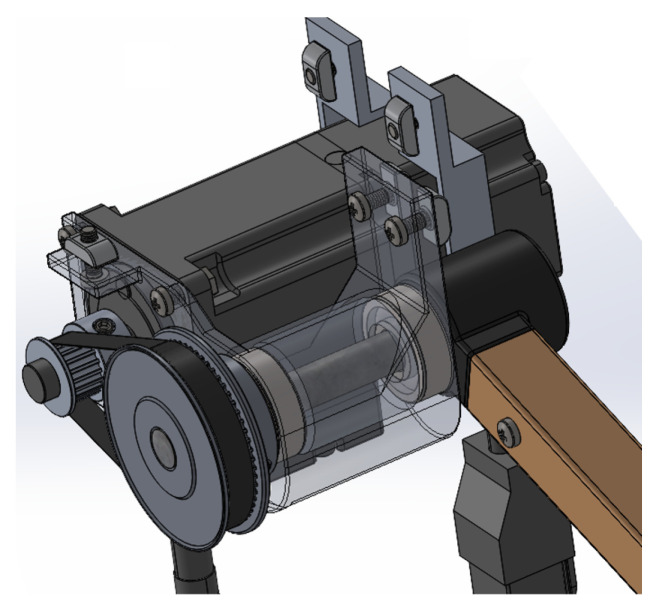
Design in SolidWorks of the Delta robot actuator coupled to an arm.

Figure 2 shows the SolidWorks design of the servomotor coupled to one of the Delta robot’s arms through a pulley and belt transmission mechanism. This system allows the motor’s motion to be efficiently transmitted to the robot’s arm, ensuring precision and control in the mechanism’s operation.

Despite its reliability and robustness, the selected servomotor is not exempt from possible failures that could affect its performance in the Delta robot. Some of the most common causes of electrical disconnection include power supply issues, such as voltage fluctuations or supply interruptions, as well as failures in wiring or connectors, which may cause disruptions in energy and signal transmission. Additionally, the Lexium LXM28 servo drive may activate protection mechanisms in the event of overcurrent, overheating, or communication failures with the controller, shutting down the motor to prevent further damage.

This type of servomotor uses a 20-bit single-turn absolute encoder for position feedback. This encoder is a device that provides a precise measurement of the shaft angle in each complete revolution of the motor. Unlike incremental encoders, this type of encoder retains the absolute position even after a power-off or reset.

Being 20-bit, it can resolve $2^{20}$ = 1,048,576 distinct positions within a single shaft revolution, which corresponds to an angular precision of approximately $0.00034^{\circ }$ per increment. A single-turn encoder only measures the position within one revolution of the shaft ($0^{\circ }$–$360^{\circ }$) without recording the number of complete rotations.

In the Delta robot, this encoder enables precise measurement of each joint’s position, which is critical for kinematics and trajectory control. Its high resolution ensures smooth and accurate movements, improving precision in manipulation and assembly tasks.

Despite its high precision and reliability, 20-bit single-turn absolute encoders can experience failures that affect position measurement and, consequently, the performance of the Delta robot. These failures may be caused by electrical, mechanical, or environmental issues, impacting the system’s correct feedback.

## 4. Design of the Fault Diagnosis System

An active fault-tolerant control system relies on an online diagnosis of faults; that is to say, it is necessary to first determine the presence of the fault, the type of fault, its size, and the time of occurrence [16]. Based on this information, some accommodation mechanisms must be activated, the control reconfiguration, or even, depending on the severity, the system stoppage. This approach requires having a fault diagnostic system that, in real time, can provide information to a supervisory system so that it can activate some corrective action mechanism.

The fault diagnostic system proposed in this research uses the robot control signals as symptom variables of the present faults. These control signals, when dealing with the malfunction of the manipulator, show significant variations as a result of their effort to maintain the desired trajectory.

A feature extraction algorithm is proposed, integrating Wavelet Scattering Networks (WSNs), Principal Component Analysis (PCA), Linear Discriminant Analysis (LDA), and Meta-Learning (ML) for robust fault diagnosis. First, WSNs are applied to the control signals. This results in an initial reduction in the data dimensionality. Subsequently, the sample size is further reduced and the separability between classes is improved, using PCA and LDA, respectively. As the last stage of the feature extraction process, machine learning of the fault patterns is implemented using ML.

**Figure 3:**
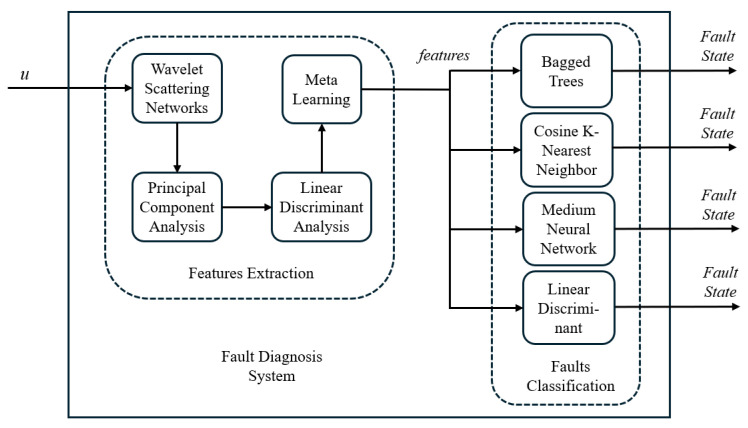
Fault diagnostic system diagram.

Once the features of the system states are obtained, they are classified in the MATLAB Classification Learner. In this work, four different classifiers are evaluated, and they were selected due to their diverse approaches to data classification. The classifiers considered were Bagged Trees (BTs), known for their ability to handle complex data by combining multiple decision trees; Cosine K-Nearest Neighbor (CKNN), which uses cosine-based similarity metrics to identify close patterns; Medium Neural Network (MNN), characterized by its ability to model non-linear relationships in data; and Linear Discriminant (LD), which offers a linear and efficient approach to class separation. This selection enables the exploration of different classification methodologies for fault diagnosis, providing a broad view of their applicability and effectiveness in this context. Figure 3 shows in a diagram the proposed fault diagnostic system.

### 4.1. Analyzed Faults

Independent simulations of three faults on the manipulator were performed, each lasting 15 s. The first fault corresponds to a breakdown in actuator 3, causing a loss of control over that joint. The second fault is located in the position sensor of motor 2, resulting in incorrect feedback of the joint movement. Finally, the third fault is of multiple types, simultaneously combining the two previous faults.

The selection of the nodes where faults are analyzed in the Delta robot has been carefully justified based on their impact on system performance and the criticality of their function within the robot’s structure. Specifically, motors and encoders have been chosen as the main components for fault analysis, as they are fundamental to ensuring precise joint movement and, consequently, accuracy in task execution.

The motors were selected because they are the primary actuators of the system, responsible for generating the robot’s movement. Any malfunction, such as loss of torque, overheating, or electrical failures, can significantly affect control stability and the robot’s ability to follow planned trajectories. On the other hand, encoders play a crucial role in position and speed feedback, ensuring the precision of each joint’s control. Faults in encoders, such as position measurement errors or signal interruptions, can compromise synchronization and the overall performance of the control system.

Moreover, the selection of these nodes is based on the frequency and relevance of failures in similar systems. Previous studies in industrial robots have shown that motors and encoders are critical points where failures commonly occur, justifying their detailed analysis to ensure a reliable fault diagnosis and potential implementation of fault-tolerant strategies.

**Figure 4:**
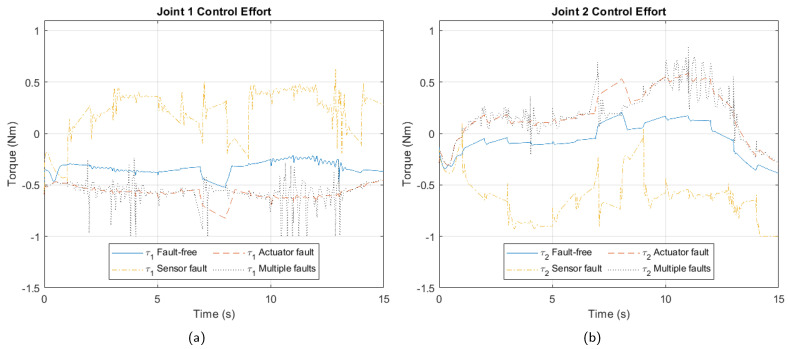

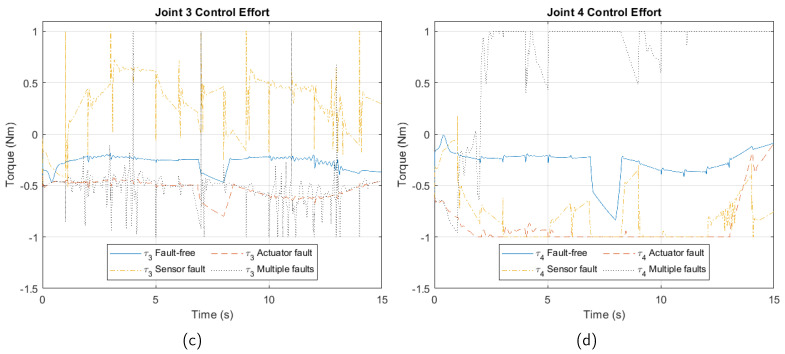
Control effort due to faults. (**a**) Joint 1. (**b**) Joint 2. (**c**) Joint 3. (**d**) Joint 4.

Figure 4 depicts joint control efforts under each fault. Readers will note that despite a fault in one joint, its effects manifest across all four arms’ control efforts, reflecting the Delta robot’s highly nonlinear closed kinematic chain.

Control signals are stored, and based on these data, features are extracted and used in MATLAB’s Classification Learner to detect and diagnose the fault state of the manipulator.

### 4.2. Feature Extraction

#### 4.2.1. Wavelet Scattering Networks

A WSN is used for the initial extraction of robust features from the control signals. WSNs are wavelet transform-based tools that allow capturing translation-invariant and deformation-stable features, which is crucial for signals affected by non-linear fluctuations.

The scattering model configuration was set with a sampling frequency of 700 Hz and an invariance scale of 21, ensuring that the extracted features retain the structural information of the signal while minimizing the effects of translations and noise. To optimize computational efficiency and reduce redundancy in the obtained coefficients, the path optimization option was enabled, improving the quality of representation without compromising class discriminability.

Subsequently, the scattering coefficients generated by the WSNs were transformed using a logarithmic function to enhance data normalization, reduce the influence of extreme values, and highlight differences between transient events and stationary signals. This transformation is crucial for improving class separation, and facilitating the identification of characteristic patterns associated with different fault states.

Additionally, the arrangement of the data in the input matrix was performed with an appropriate transposition to ensure that each row represented an independent observation, guaranteeing correct interpretation by the model. Table 1 shows the properties of the applied WSN.

#### 4.2.2. Dimensionality Reduction

To handle the still high dimensionality of the extracted features, a two-stage dimensionality reduction scheme using PCA and LDA is applied.

#### Principal Component Analysis

PCA is applied to the feature matrix obtained from WSNs, which contains the transformed representation of the input signals. The process begins with the decomposition of this matrix into its principal components, generating another matrix that contains the features projected into a new space of orthogonal dimensions.

Subsequently, a subset of these components is selected, meaning that up to 35 principal components will be retained, or, if the matrix has fewer than 35 dimensions, all available components will be used. This strategy ensures a balance between dimensionality reduction and the preservation of explained variance, allowing the retention of the most relevant information for classification without including unnecessary features that may introduce noise.

A distinctive characteristic of this implementation is that the number of selected components is defined adaptively based on the number of available dimensions in the matrix. Unlike approaches where the number of principal components is determined based on an explained variance threshold (e.g., 95% of cumulative variance), this method opts for a maximum of 35 components, providing a computationally efficient and suitable solution for the dataset used. This approach avoids issues associated with high dimensionality, such as the curse of dimensionality, and enhances the classifier’s performance by working with more representative and compact features.

#### Linear Discriminant Analysis

LDA is applied to the feature set previously reduced by PCA, with the goal of projecting the data into a new space where the separation between fault classes is maximized. This is achieved by finding linear combinations of the original features that maximize the distance between classes while minimizing the dispersion within each class, thereby facilitating the accurate classification of different fault states.

The implementation is carried out using the ‘fitcdiscr’ function, which fits a linear discriminant model to the input data. Here, the ‘pseudolinear’ discriminant type is employed, assuming linear class separation while incorporating adjustments to accommodate non-Gaussian class distributions effectively. Additionally, a Gamma parameter of 0.5 is set, introducing a regularization term to improve the numerical stability of the model and prevent overfitting when the data has high dimensionality or when the class structures are complex.

Once the LDA model is trained, the features are projected into the discriminant space using the ‘predict’ function. This operation generates a new representation of the data in which the differences between classes has been maximized. This transformation is crucial for subsequent classification, as it ensures that the data from each fault category are better separated, reducing class overlap and thereby improving the accuracy of the final classifier.

#### 4.2.3. Meta-Learning for Parameter Optimization

The ML approach allows the system to learn not only from individual data points but also through the generalization of patterns across different learning tasks, enhancing the model’s adaptability to various classification contexts.

One of the key elements in this approach is the loss function, defined as an anonymous function that evaluates the model’s performance based on the current parameters. The purpose of this function is to assess the model’s generalization capability across different data subsets, ensuring that the learned parameters remain useful in multiple contexts rather than being optimized for a single dataset.

To optimize the parameters in ML, the Sequential Quadratic Programming (SQP) algorithm is used through the ‘fmincon’ function, which aims to minimize the loss function. Specific options are set in the configuration, including ‘Display’, ‘iter’, which allows the monitoring of the optimization progress at each iteration. Additionally, in this implementation, the ‘SpecifyObjectiveGradient’, ‘false’ option is maintained, indicating that the gradient calculation is not explicitly defined and will be internally estimated by the optimizer.

Finally, the optimization is executed by calling ‘fmincon’, where the initial parameters are iteratively adjusted to minimize the loss function. No explicit constraints are imposed on the search ranges; this flexibility allows the algorithm to explore a broad region of the solution space in search of the best possible configuration. The result of this process represents the optimized version of the parameters through ML, ready to be used in the next stage of the fault diagnosis model.

This approach integrates complementary techniques that allow robust fault diagnosis. The combination of WSNs with ML ensures the extraction of significant features, while PCA and LDA allow the data to be represented efficiently, maximizing performance in the subsequent faults classification. Algorithm 1 shows the pseudocode of the feature extraction method programmed in MATLAB version R2023a.
**Algorithm 1:** Feature Extraction for Fault Diagnosis
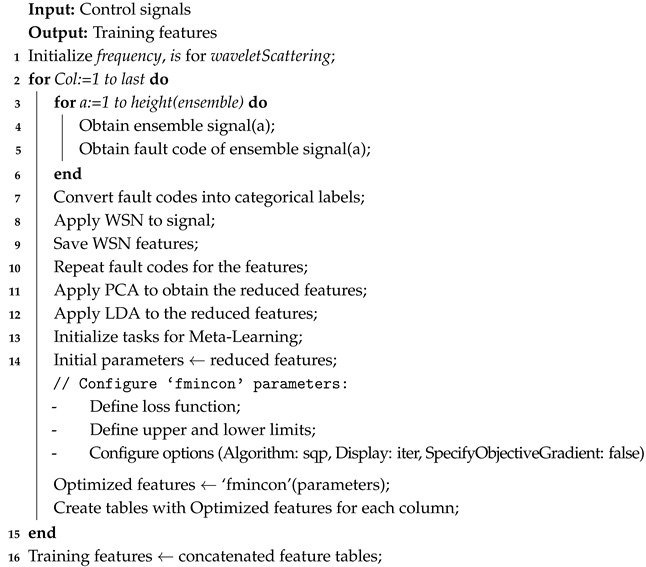


### 4.3. Faults Classification

The system fault state classification stage is implemented in MATLAB’s Classification Learner. Four algorithms are trained to evaluate their fault diagnosis capabilities. Each classifier has distinctive features that differentiate them from each other, which provides greater insight during the analysis.

#### 4.3.1. Bagged Trees

Bagged Trees (BTs), short for Bootstrap Aggregation Trees, is an ensemble learning method that reduces the effects of overfitting, which is characteristic of individual decision trees. Bootstrap aggregation, also known as bagging, is a general-purpose procedure for reducing the variance of a statistical learning method that improves the robustness and accuracy of classification tasks.

**Figure 5:**
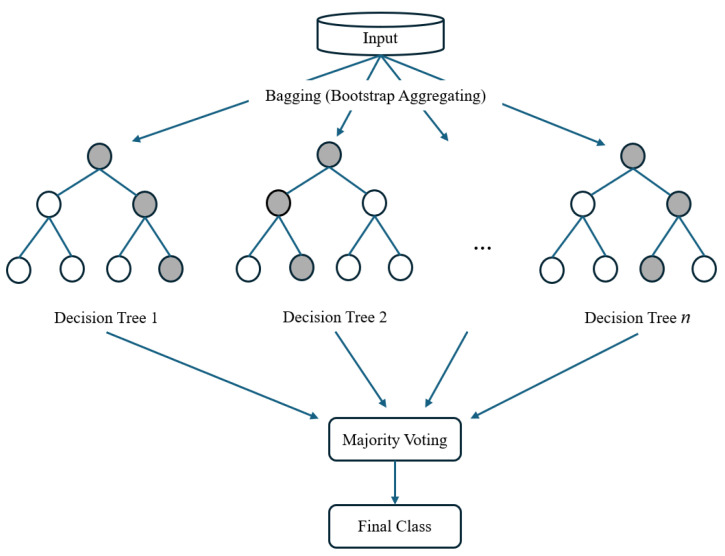
Bagged trees general structure.

Bagged Trees generate multiple subsets of the training data using random sampling with replacement. Each subset is used to train a decision tree independently on its respective subset. These trees are typically not pruned, meaning they capture intricate patterns in the data. For classification tasks, bagged trees use majority voting to combine the results of individual trees. The final prediction is the class most frequently predicted by the ensemble [30,31]. Figure 5 shows the general structure of the bagged trees.

#### 4.3.2. Cosine K-Nearest Neighbor

The Cosine K-Nearest Neighbor (CKNN) algorithm is a variant of the K-Nearest Neighbor (KNN) algorithm that uses cosine similarity, using Equation (1) as a distance metric to determine how similar two vectors are by calculating the cosine of the angle between them [32].

(1) $$\mathrm{Cosine}\;\mathrm{similarity}\;=\frac{u\cdot v}{\left\|u\|\;\|v\right\|}$$

If the similarity value is close to 1, the vectors are very similar (aligned). If it is close to 0, the vectors are orthogonal (not related). If it is negative, the vectors point in opposite directions.

The algorithm stores a labeled dataset, where each observation is represented by a feature vector and a class label. Given a new test vector, the algorithm calculates the cosine similarity between this vector and all vectors in the training set. It then selects the *K* vectors with the highest similarities. The test vector class is assigned based on the predominant class among its *K* most similar neighbors.

This algorithm requires no assumptions about the data distribution, works well with vectors representing normalized signals, and is suitable for multiclass problems. Although typically memory intensive, CKNN is a powerful tool for classifying faults in complex systems, especially when the preprocessed data have well-defined characteristic patterns.

#### 4.3.3. Medium Neural Network

Medium Neural Networks (MNN) are a category of neural networks with a moderate number of parameters and layers. These networks offer a balance between modeling capability and computational efficiency.

They are complex enough to model non-linear patterns in data, but not so large as to require huge datasets or advanced computational infrastructure. They use architectures with fewer layers and neurons compared to deep networks, which reduces the risk of overfitting and training time.

This model is a feedforward, fully connected neural network for classification. The first fully connected layer of the neural network has a connection from the network input (predictor data), and each subsequent layer has a connection from the previous layer. Each fully connected layer multiplies the input by a weight matrix and then adds a bias vector. An activation function follows each fully connected layer. The final fully connected layer and the subsequent softmax activation function produce the network’s output, namely classification scores (posterior probabilities) and predicted labels [33].

#### 4.3.4. Linear Discriminant

The Linear Discriminant (LD) classifier is a supervised classification tool based on Linear Discriminant Analysis (LDA). It is widely used for classification problems, especially when classes are linearly separable or nearly so. It is also fast, accurate, and easy to interpret.

The discriminant analysis assumes that different classes generate data based on different Gaussian distributions [33]. The goal of LDA is to find a linear combination of the dataset features that maximizes separability between classes. It does this by projecting the data into a lower-dimensional space, optimizing the relationship between interclass variance (difference between class means) and intraclass variance (dispersion within each class).

## 5. Active Fault-Tolerant Control Strategy

Fault-tolerant control can be said to exist in a control loop when one of the following exists:

- Mechanisms introducing redundancy in sensors and/or actuators.
- Adaptive strategies for reconfiguring the control law governing the loop.

**Figure 6:**
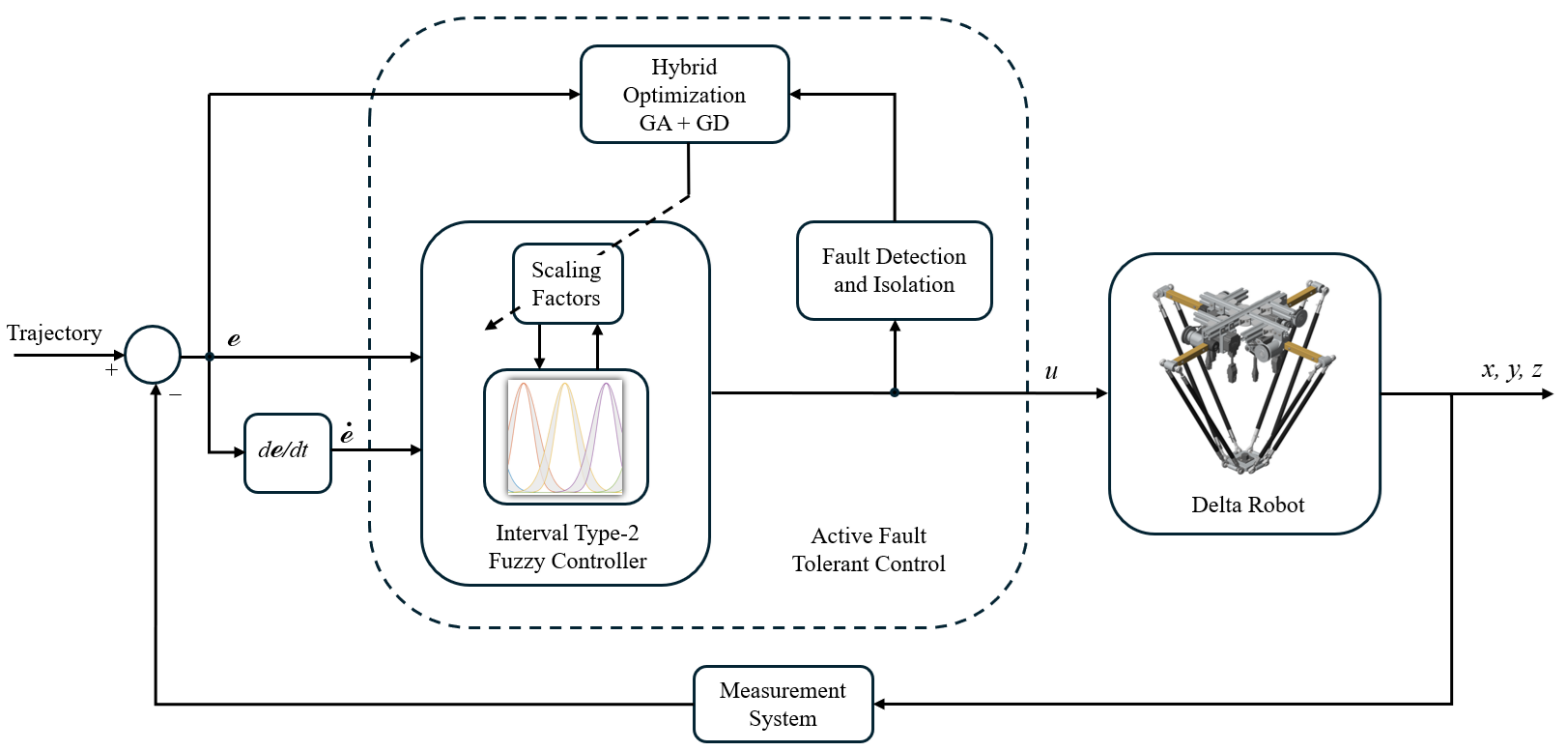
Active fault tolerant control loop diagram.

In this study, fault tolerance is implemented using the second approach. The controller is based on Interval Type-2 Fuzzy Logic (IT2FLC) with a PID structure, where the inputs are the tracking error and its derivative. The supervisor is designed using an adaptive approach based on hybrid optimization, combining genetic algorithms and gradient descent. This optimization is applied to the scaling factors of the controller. Figure 6 presents a diagram of the proposed fault-tolerant control loop.

### 5.1. Interval Type-2 Fuzzy Logic Controller

Interval type-2 fuzzy logic is distinguished by the introduction of a second-level fuzzy set, known as a type-2 fuzzy set. The region formed by the two membership functions represents the range of membership degree values for the inputs. This capability allows for a more robust modeling of the uncertainties within the controlled system. In particular, Interval Type-2 Fuzzy Sets (IT2FS) restrict the membership degrees to defined intervals, which significantly simplifies the mathematical operations required for their management.

**Figure 7:**
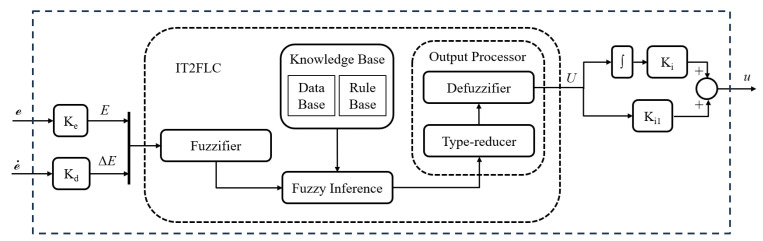
Diagram of the interval type-2 fuzzy logic based controller.

Figure 7 presents the scheme of the proposed Interval Type-2 Fuzzy Logic Controller (IT2FLC) with a PID structure. The controller receives as inputs the tracking error and its derivative. The terms $K_{e}$, $K_{d}$, $K_{i}$, $K_{i1}$ represent the scaling factors, which are used to adjust the magnitudes of the signals before entering and after exiting the fuzzy controller. These factors help to adjust the sensitivity of the controller and are essential for achieving the desired performance of the controlled process [29].

**Figure 8:**
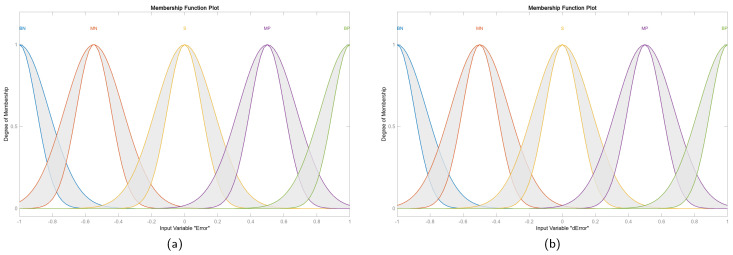
Membership functions for the inputs. (**a**) Input 1: ‘Error’. (**b**) Input 2: ‘dError’.

The IT2FS of the proposed controller are formed by five membership functions as shown in Figure 8. The rule base for inference is detailed in Table 2.

### 5.2. Genetic Algorithms

Genetic Algorithms (GAs) are optimization techniques inspired by the principles of natural selection and biological evolution. They create and evolve a population of possible solutions to a problem, where each solution is represented as an “individual” in the form of a string (chromosome). Throughout the optimization process, individuals undergo genetic operations like selection, crossover, and mutation, simulating the process of evolution. Over successive generations, the fittest solutions are selected and evolved, improving the population’s quality and approaching the optimal solution [34].

GAs are particularly effective in optimization problems with multiple variables, non-linear functions, and complex search spaces, as they do not require derivatives or problem-specific information. Furthermore, they are able to escape from local optima, making them a robust tool for exploring large solution spaces.

The optimization is performed using MATLAB’s ‘ga’ function, which enables finding an optimal set of parameters through an evolutionary process inspired by natural selection. This approach is particularly useful when the search space is complex, and a well-defined gradient function is not available.

A population size of 100 individuals is defined, ensuring a broad exploration of the solution space in each iteration. The number of generations is set to 50, determining how many times the evolutionary process will iterate to improve the solution. The crossover rate is fixed at 0.8, meaning that 80% of the new generation will be formed from combinations of previous solutions, promoting genetic diversity and a thorough exploration of the search space. Additionally, an adaptive mutation function is employed, which dynamically adjusts the mutation magnitude based on the feasibility of the generated solutions.

Finally, the optimization is executed by calling the ‘ga’ function, which seeks to minimize the cost function by adjusting the four scaling factors of the controller. The optimal solutions are found within specified limits, restricting the search to feasible values. The optimization result returns a vector containing the optimal parameters found using the GA.

### 5.3. Gradient Descent

The Gradient Descent (GD) method is an optimization algorithm used to minimize differentiable functions. It starts from an initial solution and performs iterative adjustments following the direction of the gradient of the objective function, which indicates the steepest slope toward decreasing function value. The size of the adjustment step is determined by a parameter called the “learning rate”. As the algorithm progresses, it approaches a point where the gradient is zero, which corresponds to a local or global minimum of the function [35].

GD is efficient for finding solutions in problems where the objective function is smooth and differentiable, and its speed of convergence makes it a very popular method in function optimization.

In this application, the GD is performed with MATLAB’s ‘fmincon’ function, which seeks to minimize the defined cost function. This function evaluates the system’s performance based on the fault-state position, the fault-free position, the control effort, and the previous scaling factors, ensuring that the optimization is carried out based on criteria relevant to fuzzy control.

To ensure efficient tuning, the optimization algorithm is set to ‘interior-point’, a robust method for nonlinear constrained problems. Additionally, parallel execution is enabled, allowing for faster optimization by distributing computations across multiple processing cores. Extremely low tolerances are also set for the cost function evaluation (OptimalityTolerance = 1 × 10^−8^, StepTolerance = 1 × 10^−8^, FunctionTolerance = 1 × 10^−8^), ensuring that the refinement process is conducted with maximum precision and preventing premature termination of the optimization.

Finally, the optimization is executed by calling ‘fmincon’. This process iteratively adjusts the parameters within the specified limits, aiming to reduce the cost function (detailed in Section 5.6) until a refined optimal solution is reached. The final result is the set of scaling factors optimized using GD, representing the most precise and fine-tuned version of the fuzzy logic-based controller.

### 5.4. Advantages of Hybrid Optimization for Control Law Reconfiguration

Combining GAs and GD into a hybrid optimization approach offers several advantages for control law reconfiguration:

- Enhanced exploration and exploitation: GAs are excellent for exploring the solution space, generating multiple initial solutions, and escaping local optima thanks to mutation and recombination operators. However, they can be less accurate in the final exploitation phase. GD, on the other hand, excels in exploitation, quickly converging towards an optimum once near the solution. The combination of both allows a balance between exploration and exploitation, ensuring a more complete and effective search for the optimal solution.
- Escape capacity from local optima: GAs diversify the solution population, reducing the likelihood of being trapped in local optima. GD then refines promising solutions found by GAs, ensuring higher precision.
- Flexibility for complex problems: The combination addresses highly nonlinear problems, such as those in Delta-type parallel robots, where the objective function is complex and has many local optima. The synergy between the two methods facilitates the adaptation of the system to changes or failures in real time.
- Faster convergence and computational efficiency: Using GAs to generate a good initial solution, followed by GD for refinement, results in a more time-efficient and resource-efficient optimization process. As detailed in Section 5.5, the hybrid approach achieves convergence in approximately 0.52 s per cycle, leveraging GA’s global search (O(9 × 10^7^) operations) and GD’s rapid local refinement (O(5.4 × 10^6^) operations). This balance ensures efficient resource use while maintaining real-time applicability, as the total computation time occupies less than 4% of the 15 s trajectory cycle, with potential for further optimization on dedicated hardware.

The hybrid approach leverages the strengths of both methods, providing a robust and effective optimization strategy for control law reconfiguration. This is especially valuable for applications like the Delta robot, where adaptability and precision are essential to maintain robot performance under fault conditions. Algorithm 2 exposes the pseudocode used to implement the optimization.
**Algorithm 2:** Pseudocode for hybrid fault-tolerant controller optimization. **Input**: Original Scaling Factors, Tracking Error, Control Effort **Output**: New scaling factors **_1_** Define lower and upper limits for the scaling factors optimization; **_2_** Configure options for GAs (Display: iter, UseParallel: true, PopulationSize: 100, MaxGenerations: 50); **_3_** Define cost function: FuzzyCostFunction (Tracking Error, Control Effort, Original Scaling Factors); **_4_** Execute global optimization with GAs; **_5_** Save scaling factors obtained with GA optimization; // Use scaling factors from GA optimization as starting point for DG: **_6_** Define DG Initial values ← GA scaling factors; **_7_** Configure options for DG (Display: iter, Algorithm: interior-point, UseParallel: true); **_8_** Execute local optimization with DG; **_9_** New scaling factors ← results from optimization with DG;


### 5.5. Computational Complexity of Hybrid Optimization

The hybrid optimization framework proposed in this study integrates GAs and GD to achieve robust control law reconfiguration. Understanding the computational complexity of these methods is critical for assessing their feasibility in real-time robotic applications, particularly for Delta robots requiring rapid response times. Below, we analyze the theoretical complexity and provide empirical execution times based on the implementation in MATLAB R2023a on a standard computing platform.

#### 5.5.1. Genetic Algorithm Complexity

GAs operate by evolving a population of solutions over multiple generations, involving operations such as selection, crossover, and mutation. The computational complexity of GAs can be expressed as O(G × P × C), where G is the number of generations, P is the population size, and C is the cost of evaluating the fitness function for each individual. In this study, G = 50 and P = 100, as specified in Section 5.2. The cost function, detailed in Section 5.6, involves calculating the tracking error, control effort, and scaling factor variations across N = 15,001 time steps and M = 3 axes (X, Y, Z), resulting in a complexity of O(N × M) per evaluation. Thus, the total complexity of GAs is approximately O(20 × 100 × 15,001 × 3) = O(9 × 10^7^) operations. GA optimization averages 0.41 s per fault scenario, reflecting an efficient implementation.

#### 5.5.2. Gradient Descent Complexity

GD iteratively adjusts the scaling factors by following the gradient of the cost function until convergence. Its complexity is O(I × D × F), where I is the number of iterations, D is the number of parameters to optimize (D = 4, corresponding to $K_{e}$, $K_{d}$, $K_{i}$, $K_{i1}$), and F is the cost of computing the gradient. The gradient computation involves evaluating the cost function (O(N × M)) and its partial derivatives, approximated numerically in this implementation, leading to a per-iteration complexity of O(N × M × D). With tolerances set to 1 × 10^−8^ (Section 5.3), GD typically converges in I ≈ 30 iterations for the given initial conditions from GAs, resulting in a total complexity of O(30 × 4 × 15,001 × 3) = O(5.4 × 10^6^) operations. GD execution averages 0.26 s per fault scenario, leveraging efficient gradient approximations and the interior-point algorithm.

#### 5.5.3. Hybrid Approach and Real-Time Feasibility

The hybrid approach first employs GAs for global exploration, followed by GD for local refinement, yielding a combined complexity of O(9 × 10^7^ + 5.4 × 10^6^) ≈ O(9.5 × 10^7^) operations per reconfiguration cycle. The total execution time averages 0.52 s (0.41 s for GA + 0.26 s for GD − 0.15 s overlap due to initialization efficiency), as summarized in Table 3. This duration represents a moderate increase over GAs alone but delivers significant improvements in controller performance, balancing exploration and refinement. This 0.52 s duration suits real-time use, as reconfiguration at the end of each 15 s cycle occupies under 4% of the cycle time. In high-speed applications requiring sub-second responses, optimizations such as reducing population size (e.g., P = 50) or deploying on specialized hardware (e.g., FPGA or DSP) could further decrease computation time to below 0.3 s, aligning with stringent industrial requirements.

However, reducing P may compromise solution accuracy, while hardware acceleration increases implementation cost, necessitating a trade-off between speed and precision tailored to specific use cases. These considerations highlight the need to balance computational efficiency with control performance in industrial deployments.

### 5.6. Cost Function for Scaling Factors Optimization

In this study, the optimization of scaling factors ($K_{e}$, $K_{d}$, $K_{i}$, $K_{i1}$) is performed using a cost function that evaluates the system’s performance. The cost function considers three fundamental aspects: tracking error, control effort, and prior scaling factors.

The cost function *J* penalizes tracking errors between fault-free and faulty positions, control effort for trajectory regulation, and abrupt scaling factor changes. The general Equation (2) of the cost function is expressed as a weighted combination of these factors, where α, β, and δ are coefficients that balance the relative importance of the tracking error, the control effort and scaling factor variations.

(2) $$J=\alpha \cdot E_{\mathrm{tracking}}+\beta \cdot u_{\mathrm{control}}+\delta \cdot K_{scaling}$$

The term $E_{\mathrm{tracking}}$ is the accumulated tracking error, which represents the quadratic difference between the fault-free trajectory and the faulty trajectory. It is obtained by Equation (3), where *N* is the total number of observations; *M* represents the coordinate space X,Y,Z; $P_{fault-free,ij}$ is the value of the fault-free position at the instant *i* and axis *j*; $P_{actual,ij}$ is the value of the faulty position at the instant *i* and axis *j*.

(3) $$E_{tracking}=\sum_{i=1}^{N}\sum_{j=1}^{M}{\left(P_{fault-free,ij}-P_{actual,ij}\right)}^{2}$$

Excess of control effort under fault $u_{\mathrm{control}}$ is obtained with Equation (4), where *L* is the total number of values in the control signal; $U_{k}$ is the control effort in index *k*; $U_{umbral}=1$ represents the upper limit of the acceptable range. This term is intended to minimize excessive energy or power usage by the system, encouraging control solutions that are more efficient in terms of applied effort.

(4) $$u_{control}=\sum_{k=1}^{L}max(0,|U_{k}{\left|-U_{umbral}\right)}^{2}$$

The scaling factors sensitivity penalty $K_{scaling}$ measures the abrupt changes between the current and previous scaling factors. It is calculated with Equation (5), where *Q* is the amount of scaling factors; $K_{m}$ represents the value of the current scaling factor in index *m*; $K_{previo,m}$, is the value of the previous scaling factor in index *m*.

(5) $$K_{scaling}=\sum_{m=1}^{Q}{\left(K_{m}-K_{previo,m}\right)}^{2}$$

To balance the objectives, the weighting parameters α, β, and δ are used, which can be adjusted depending on the priorities of the system. In this case, the values α=0.6, β=0.3, and δ=0.1 are assigned, giving greater relevance to the tracking error.

The goal of this optimization is to find the optimal values of $K_{e}$, $K_{d}$, $K_{i}$, $K_{i1}$ that minimize the value of the cost function *J*. By minimizing *J*, a balance is ensured between minimizing the tracking error, limiting the control effort and avoiding sharp changes in the scaling factors, providing adequate performance for the reconfiguration and efficiency of the control system.

### 5.7. Degradation Levels and Residue Analysis

In a fault-tolerant control system, it is common to define degradation levels for the system when a fault occurs. These levels enable the evaluation and classification of the fault’s impact on system performance, as well as the effectiveness of the fault-tolerant control in managing it. Additionally, the degradation levels help identify the severity of the fault and the degree of functionality that the system can maintain under such conditions. The most common are as follows:

- Normal or fault-free level: At this level, the system is operating correctly, with no faults detected, and the controller is operating in its nominal mode. Performance is optimal and the system meets all operating requirements without restrictions.
- Minor or partial fault level: A minor fault is detected in the system, but the controller can efficiently compensate for it. At this level, the degradation in performance is minimal and does not significantly affect the system’s objectives. The system can operate close to its normal specifications, albeit with a slight decrease in accuracy or stability.
- Moderate fault level: The fault is more significant and the system suffers a noticeable degradation in performance. However, the fault-tolerant controller adjusts its behavior to maintain acceptable functionality. Some specifications, such as response time or accuracy, may be compromised, but the system remains operational, albeit with degraded performance.
- Critical fault level: At this level, the failure severely impacts the system, and the controller can only maintain very limited functionality. Performance degradation is high, and some or more of the control objectives may be sacrificed. The system can still operate in a degraded emergency mode, but with greatly reduced capabilities.
- Irreversible or catastrophic fault level: This is the most severe level, where faults cannot be compensated for and the system may stop functioning completely. The controller may enter a fail-safe mode or shut down the system to prevent further damage. At this level, system recovery may require manual intervention or physical repair.

#### Residual Analysis in Trajectory Tracking

In this context, the term residual refers to the difference between the path traveled by the robot under normal conditions and under a fault state. This residual reflects the level of degradation present in the system. To quantify the levels of degradation, the performance indices described in Equations (6) and (7) are defined. The Integral Square Error (ISE) calculates the accumulated squared error over time, while the Integral of Time-weighted Absolute Error (ITAE) measures the time-weighted absolute error, providing a more detailed assessment of the faults impact based on their duration.

(6) $$ISE=\int_{0}^{T}e^{2}\left(t\right)dt$$

(7) $$ITAE=\int_{0}^{T}t\left|e(t)\right|dt$$

In both equations, e(t) represents the error at time *t*, defined as the difference between the fault-free and faulted end-effector positions, and *T* is the evaluation period’s final time.

To facilitate the residual classification according to its degradation level, it is proposed to normalize the results of the performance indexes using Equation (8). In this formula, *X* represents the original values of the performance indices, $X_{min}$ is defined as zero, indicating the absence of faults, and $X_{max}$ corresponds to the level of irreversible failure.

(8) $$X_{normalized}=\frac{X-X_{min}}{X_{max}-X_{min}}$$

In the analyzed Delta robot, the irreversible fault level is determined from the values presented in Table 4, where the error exceeds the tolerable threshold for proper trajectory tracking.

Based on the metrics described and considering the results of various simulations of the robot in its different failure states, the classification criteria for the degradation levels of the Delta manipulator under study are established in Table 5. These criteria are defined based on normalized values to allow a consistent and comparative assessment.

**Figure 9:**
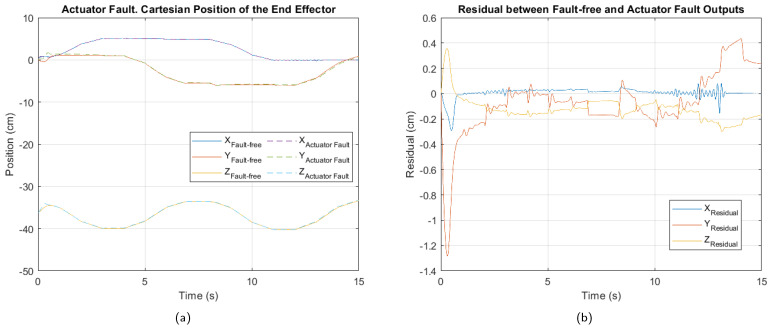
Actuator 3 fault. (**a**) Cartesian position of the end effector. (**b**) Residual between fault-free and actuator fault outputs.

**Figure 10:**
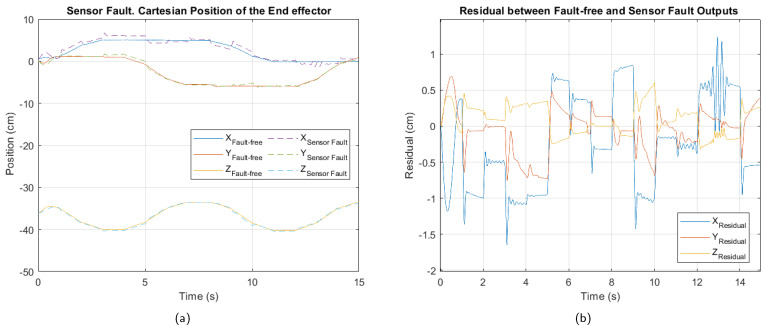
Sensor fault. (**a**) Cartesian position of the end effector. (**b**) Residual between fault-free and sensor fault outputs.

**Figure 11:**
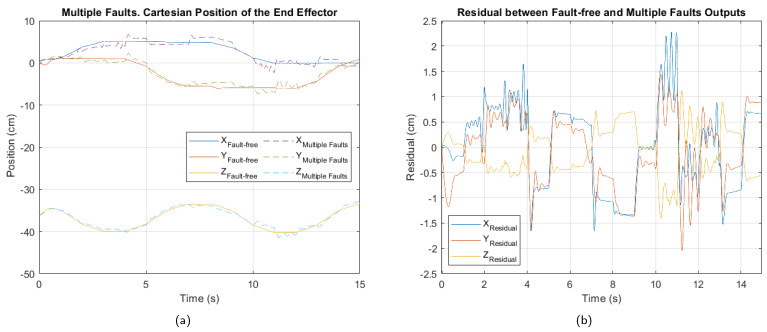
Multiple fault. (**a**) Cartesian position of the end effector. (**b**) Residual between fault-free and multiple faults outputs.

In Figure 9, Figure 10 and Figure 11, the cartesian positions of the end effector and the corresponding residuals under actuator, sensor, and multiple faults, respectively, are shown. These responses were obtained with the interval type 2 fuzzy controller, prior to the implementation of the fault-tolerant strategy. The negative impact of the multiple faults on the trajectory tracking is easily appreciated, demonstrating that the control system is unable to adequately compensate for the malfunction. In contrast, in the event of actuator failure, the magnitude of the deviations is considerably smaller, suggesting a reduced impact, as well as greater robustness of the controller against this type of fault.

Table 6, Table 7 and Table 8 show the values of the performance indices for each failure analyzed. According to the result of the normalized averages and the classification criteria established for the degradation levels, the actuator fault is of a minor type, the sensor fault is at a moderate level, while the multiple fault corresponds to a critical level.

## 6. Results and Discussion

### 6.1. Fault Diagnosis

The fault diagnosis phase utilized 15,001 initial samples per joint and fault state. Additionally, simulations were repeated for four different trajectories, generating a large dataset, of which 70% was used for training and 30% for testing. The training process utilized a 5-fold cross-validation.

In order to evaluate the effectiveness of the proposed feature extraction algorithm, the influence of each tool involved in fault classification was progressively analyzed. Initially, WSNs were combined with PCA. Subsequently, LDA was integrated into this combination. Finally, to complete this study, ML was added, thus allowing a comprehensive evaluation of the impact of each tool on the performance of the algorithm.

Table 9 presents the training and testing results for classifiers using features obtained from different combinations. The nomenclatures used are as follows Bagged Trees (BTs), Linear Discriminant (LD), Medium Neural Network (MNN), and Cosine K-Nearest Neighbors (CKNNs).

From the feature extraction combination perspective, WSN + PCA yields moderate training performance but lower test-phase accuracy. This suggests that while some relevant features are captured, they are insufficient for consistently generalizing faults.

The addition of LDA significantly improves performance in both training and testing, with test results ranging from 91.1% to 98.2%. LDA enhances class separability, resulting in robust performance. However, the better test performance compared to training indicates some overfitting introduced by LDA due to its projection of data into lower-dimensional spaces.

Adding Meta-Learning yields 100% accuracy for all classifiers in both training and testing phases. This indicates the model can perfectly fit the data and generalize effectively due to the high quality of the extracted features. Meta-Learning optimizes the hyperparameters and improves the adaptation of the model to the particularities of the faults.

From a classifier perspective, the results show solid performance across the board, especially when high-quality features are provided. Bagged Trees stand out for their stability and ability to handle overfitting introduced by LDA. Table 10 highlights the training times and characteristic sizes of classifiers trained with the full feature extraction combination. Based on these results, the LD model appears most suitable for physical implementation.

**Figure 12:**
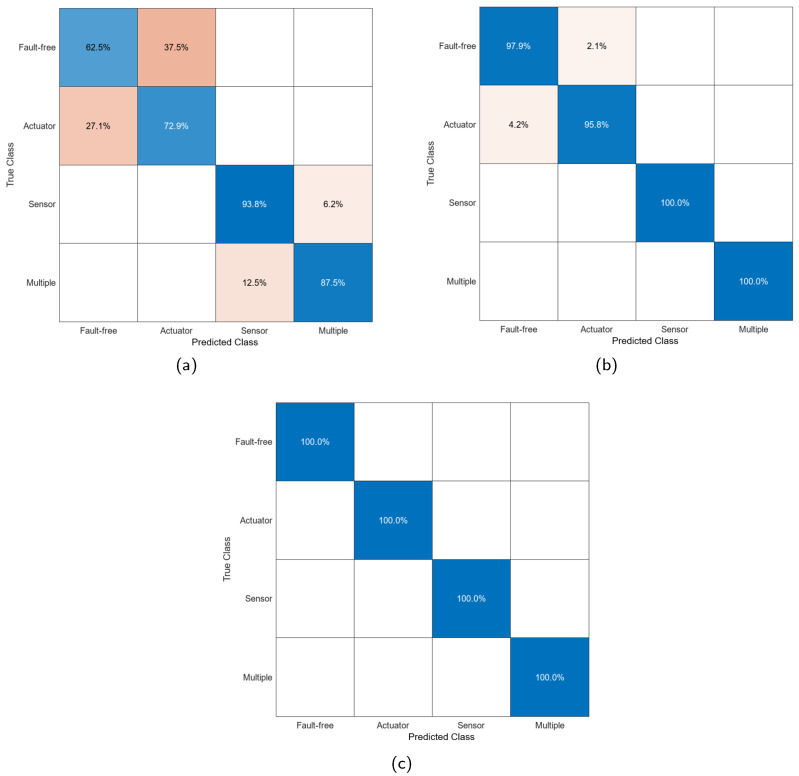
Confusion matrices from the test phase of the Linear Discriminant model. (**a**) Features extracted with WSN + PCA. (**b**) Features extracted with WSN + PCA + LDA. (**c**) Features extracted with WSN + PCA + LDA + ML.

Figure 12 presents confusion matrices from the test phase of the Linear Discriminant model for each feature extraction source. The matrices of this model are the only ones presented, taking into account their classification results and the characteristics of the algorithm. The matrices reveal that ‘Fault-free’ and ‘Actuator’ states are the hardest to classify, especially with WSN + PCA and WSN + PCA + LDA. Sensor fault is identified with the highest certainty, while multiple fault shows strong diagnostic results, particularly after incorporating LDA.

It is important to clarify the fact that the placement of faults in degradation levels does not directly influence detection success. This is because fault diagnosis is based on features extracted from control signals, while degradation levels are classified based on residuals in the end-effector’s output position.

After completing the training and testing of the classifiers, several simulations were conducted to evaluate the processing times of the proposed diagnostic system. Table 11 presents the times obtained during the signal preprocessing for feature extraction, as well as those corresponding to each analyzed classifier.

The diagnostic system’s processing times range from 68 ms to 93 ms, depending on the classifier, making them suitable for implementation as diagnosis occurs at the end of each robot trajectory cycle. Background execution reduces the need for ultra-low response times, enabling deployment on embedded hardware without performance trade-offs. Moreover, MATLAB’s interpreted environment inflates these times; optimized implementations on high-performance microprocessors or DSPs should significantly reduce them, enhancing real-world applicability.

### 6.2. Fault-Tolerant Control

The implementation of the active fault-tolerant control strategy demonstrates both effectiveness in mitigating faults and computational feasibility for practical deployment. As outlined in Section 5.5, the hybrid optimization process, requiring approximately 0.52 s per reconfiguration, aligns with the 15 s trajectory cycles of the Delta robot, ensuring that control adjustments can be applied without interrupting operational continuity. Below, we present the reconfigured scaling factors and their impact on trajectory tracking performance across the analyzed fault scenarios.

By applying the proposed active fault-tolerant control strategy, the reconfigured scaling factors shown in Table 12 were obtained. Their application allows the signals entering and exiting the IT2FLC to be modified as effectively as possible to address the faults.

**Figure 13:**
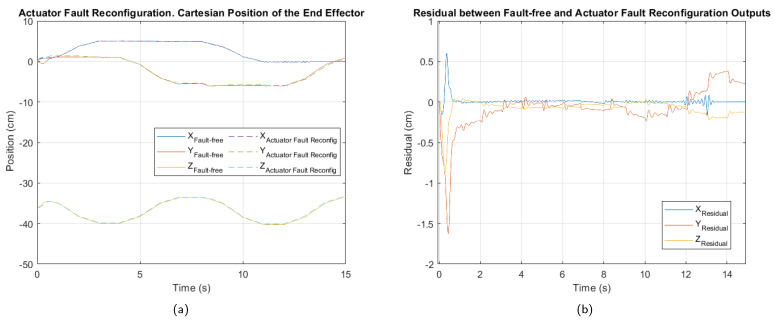
Control reconfiguration for the actuator 3 fault. (**a**) Cartesian position of the end effector. (**b**) Residual between fault-free and control reconfiguration outputs.

Regarding the actuator fault, although its impact on trajectory tracking is minor, Figure 13 shows that after controller reconfiguration, it was further reduced. While the residual indicates that some tracking precision issues persist, the system operates closer to normal conditions under this fault.

The results in Table 13 show that normalized performance index values slightly decrease compared to those before the control reconfiguration. The ISE exhibits relatively low values across all three axes, with a normalized average of 0.06. This suggests that the accumulated error over time is small, indicating that the system remains capable of efficiently correcting the trajectory despite the fault.

Notably, the X axis shows the lowest ISE value (0.03), suggesting that the system’s dynamics in this coordinate are less affected by the fault. Although the system experiences a slight level of degradation, the results demonstrate that the fault-tolerant control strategy has been effective in mitigating the impact of the actuator fault.

**Figure 14:**
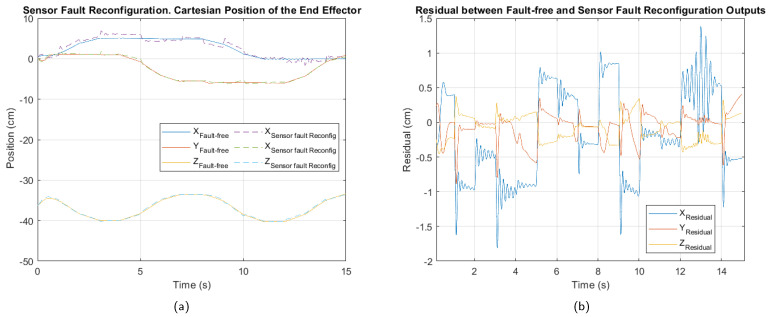
Control reconfiguration for the sensor fault. (**a**) Cartesian position of the end effector. (**b**) Residual between fault-free and control reconfiguration outputs.

For the sensor fault, Figure 14 shows the cartesian position and residual after applying fault-tolerant control. The trajectory tracking in the presence of the fault shows reduced deviation. The residual indicates that the X-axis position component is the hardest to adjust after controller reconfiguration.

The performance index values in Table 14 show that the fault-tolerant control strategy has partially mitigated the impact of the sensor fault. However, the absolute values remain higher than those observed in the case of the actuator fault, confirming that a sensor fault has a more severe impact on the overall system performance.

In particular, the high ISE value in the X axis suggests greater error accumulation compared to the other axes, while the ITAE, with a value of 62.63, indicates a persistent error over time in this coordinate. Although the values still reflect a moderate level of degradation, the observed reduction compared to the pre-reconfiguration condition demonstrates an improvement in the system’s response.

**Figure 15:**
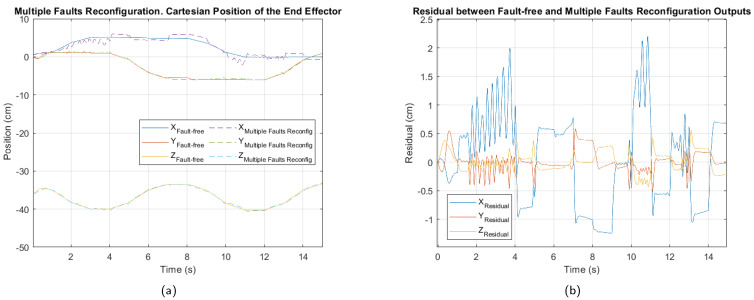
Control reconfiguration for the multiple fault. (**a**) Cartesian position of the end effector. (**b**) Residual between fault-free and control reconfiguration outputs.

The manipulator’s response after the controller reconfiguration in the presence of a multiple fault, shown in Figure 15, indicates a significant reduction in fluctuations of the end-effector positioning. This suggests that the fault-tolerant control strategy has successfully improved system stability despite the combined actuator and sensor faults. However, the overall performance impact remains greater than in cases of individual faults, highlighting the complexity of managing multiple simultaneous faults.

Comparing the results in Table 15 with those obtained for individual faults, it is evident that performance remains lower, particularly along the X axis. Nevertheless, the normalized performance index values have shifted from critical levels to moderate fault levels, confirming that the reconfiguration strategy has been effective in mitigating system degradation in the presence of multiple faults.

**Figure 16:**
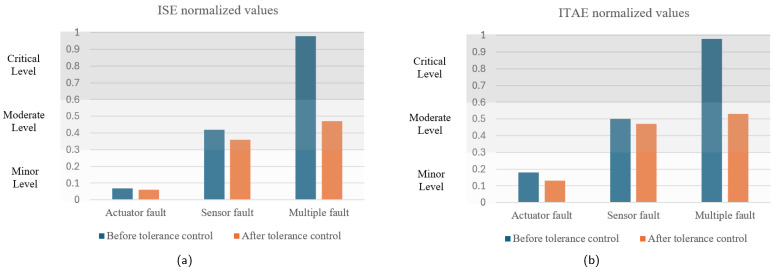
Comparison of normalized performance indices. (**a**) ISE. (**b**) ITAE.

Figure 16 compares normalized performance indices before and after the application of the fault-tolerant control strategy. These results highlight the effectiveness of the proposed active fault-tolerant strategy in mitigating the effects of faults.

The combination of an appropriate fault diagnosis phase and the proposed controller reconfiguration strategy enables the studied Delta robot to maintain stable operation, with degradation levels ranging from minor to moderate, even when faced with faults that would otherwise critically impact trajectory tracking performance.

#### Ablation Analysis

To evaluate the contribution of each technique in the proposed hybrid optimization, an ablation experiment was conducted, individually removing the algorithm components. Two experiments were performed: in the first, GD was removed, allowing the scaling factors to be determined solely by GAs; in the second, the GA was removed, leaving GD to perform the entire optimization from the initial configuration. The scaling factors obtained in the first experiment are presented in Table 16, while Table 17 shows the corresponding performance indexes.

With the second experiment, the results presented in Table 18 and Table 19 were obtained.

The results of the ablation experiment indicate that optimization using GAs achieves a better adjustment of the scaling factors compared to GD when used independently. This suggests that the optimization problem exhibits a complex landscape with multiple local optima, where the global exploration capability of GAs enables the discovery of higher-quality solutions.

In contrast, the exclusive use of GD, although it partially improves fault-tolerant control, is limited by its local nature, preventing it from reaching optimal configurations when the initialization is not adequate. However, despite GAs outperforming GD in terms of performance, neither method used independently achieves the results obtained with the hybrid optimization, highlighting the importance of combining both techniques.

These results underscore the value of a hybrid strategy, where GAs explore promising search space regions and GD refines solutions, optimizing both efficiency and stability in fault-tolerant control.

### 6.3. Stability Analysis of the Fault-Tolerant Control System

To verify the stability of the fault-tolerant control system, Lyapunov exponents were used. This approach evaluates how small initial perturbations evolve over time. A negative maximum Lyapunov exponent indicates that nearby trajectories converge, signifying asymptotic stability. In contrast, a positive maximum Lyapunov exponent indicates divergence, which is characteristic of instability or even chaotic behavior [28,36].

The maximum Lyapunov exponents were calculated in different scenarios to verify the stability of the control system. The first analysis was performed on the fault-free trajectory, introducing a 0.5 cm variation in the initial position of the end effector for each coordinate axis. The second analysis was carried out considering the trajectory in the presence of a multiple fault, thus evaluating the worst condition analyzed. Finally, the third study evaluated the trajectory under the multiple fault, but after reconfiguring the active fault-tolerant controller, in order to confirm the stability of the system following the controller’s transition. The procedure for obtaining the Lyapunov exponents is detailed in Algorithm 3.

**Figure 17:**
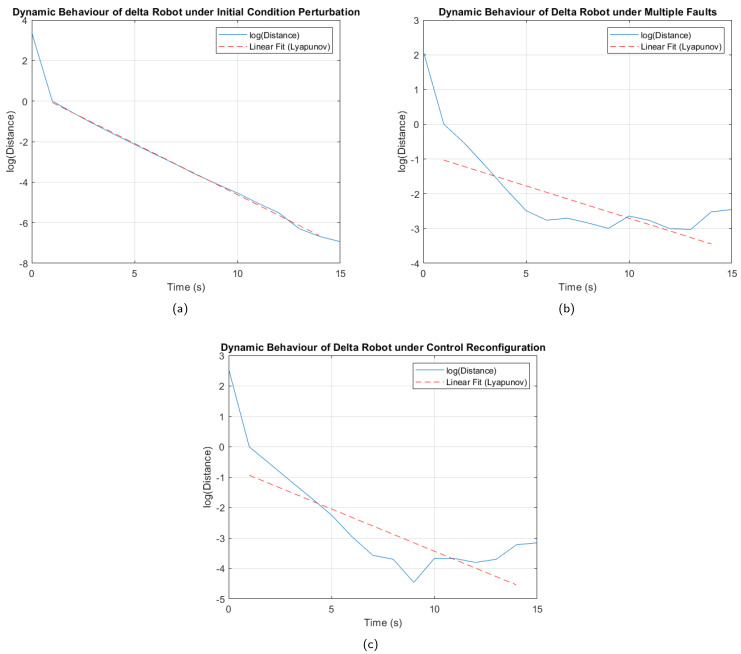
Logarithmic analysis. (**a**) Initial condition perturbation. (**b**) Trajectory under multiple faults. (**c**) Trajectory under multiples faults after control reconfiguration.

As a result of the tests carried out, Figure 17 was obtained, where it can be seen that, under the perturbation of initial conditions, without the presence of faults (Figure 17a), the curves maintain parallelism and show a negative slope, confirming stability in the system. In the case of Figure 17b, although there are oscillations in the logarithm curve, the decreasing trend is not interrupted, which indicates that the system is stable under the multiple fault analyzed. A similar behavior is observed in Figure 17c, obtained after the controller reconfiguration, where the curve represented in blue shows a consistent decreasing trend, even with fewer oscillations than those in Figure 17b, confirming improved stability.
**Algorithm 3:** Pseudocode for obtaining Lyapunov exponents.
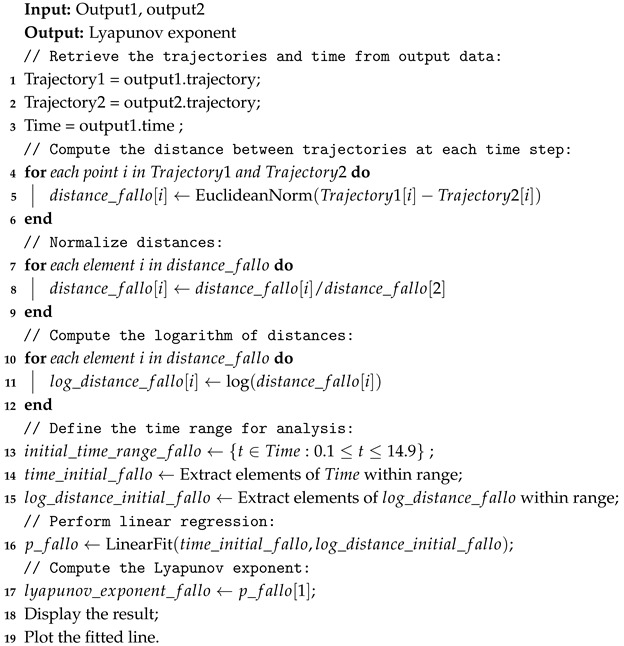


The results in Table 20, showing negative Lyapunov exponents, confirm the stability of the control system even under adverse conditions, such as the analyzed faults. Furthermore, the smaller oscillations and smoother trajectory observed in Figure 17c compared to Figure 17b, along with the corresponding Lyapunov exponent, indicate that the controller reconfiguration not only preserves stability but also significantly improves the system’s robustness, enhancing its ability to handle critical scenarios.

### 6.4. Comparison with Another Fault-Tolerant Control Method

In order to establish a comparison with the proposed method, a fault-tolerant Sliding Mode control strategy was implemented. This control strategy operates continuously by designing a sliding surface to constrain the system’s dynamics to remain on that surface. In this case, the controller generates control signals that drive the system to “slide” toward the desired behavior [37,38].

The sliding surface is typically defined in terms of the error and its derivative. The basic form of the sliding surface chosen for this application is shown in Equation (9), where *e* is the error between the reference and the output, $\dot{e}$ is the derivative of the error, and λ represents the fitting parameter of the sliding surface.

(9) $$S=\dot{e}+\lambda e$$

The initial control law is used as Equation (10), being *u* the control signal, *k* the positive gain that ensures adequate performance, and sgn(S) the sign function.

(10) u=−ksgn(S)

In a fault-tolerant Sliding Mode controller, the goal is to adjust or modify the control law when a fault is detected, maintaining stability and performance despite system degradation. One reconfiguration option is to modify λ to adjust the convergence rate toward the sliding surface. For this application, λ is adjusted dynamically as a function of the error, using Equation (11), where $\lambda_{0}$ represents the nominal value, α is the adjustment coefficient, and ∥e(t)∥ represents the modular value of the error.

(11) $$\lambda \left(t\right)=\lambda_{0}+\alpha ∥e\left(t\right)∥$$

Table 21, Table 22 and Table 23 reflect the comparison of the ISE performance index between the Sliding Mode method and the proposed hybrid optimization. For the first two fault scenarios, the Sliding Mode method achieves superior performance for individual axes in some cases. However, it fails to maintain consistent performance across all three coordinate axes. Consequently, the end effector deviates significantly from the desired trajectory under fault conditions with this method.

In the three fault scenarios analyzed, the hybrid optimization strategy presented in this study achieves lower errors overall, suggesting that this approach is more effective in minimizing the impact of faults on the robotic system.

## 7. Conclusions

This study proposed a comprehensive active fault-tolerant control strategy for Delta-type parallel robots, integrating an advanced fault diagnosis system with a reconfigurable control mechanism. By combining Wavelet Scattering Networks, Principal Component Analysis, Linear Discriminant Analysis, and Meta-Learning, the developed fault diagnosis system demonstrated high reliability and accuracy in detecting and classifying various types of faults.

The hybrid optimization framework, blending Genetic Algorithms and Gradient Descent, enabled real-time reconfiguration of an Interval Type-2 Fuzzy Controller, markedly boosting robot performance under faults. The results confirm that the proposed strategy effectively mitigates the impact of the actuator, sensor, and multiple faults, maintaining stable trajectory tracking and reducing critical degradation to moderate levels.

Furthermore, the proposed methodology contributes to the field of robotics by offering a scalable and adaptable fault-tolerant control solution applicable to other parallel manipulator architectures.

### Future Work

Future work will expand the fault diagnosis system to address intermittent and evolving faults, leveraging deep learning techniques such as convolutional neural networks. Incorporating real-time adaptive learning will enable the system to continuously update its fault detection and classification models, improving robustness in dynamic industrial environments. Additionally, exploring the integration of redundant sensory networks and predictive maintenance algorithms could further enhance the reliability of Delta robots in critical applications.

Experimental validation in real-world industrial settings will be a key focus to assess the system’s scalability and practical implementation. Moreover, optimizing computational efficiency to ensure real-time execution on embedded robotic platforms remains an important objective for future development. Scaling the hybrid approach to larger populations or faster hardware could further meet sub-second industrial demands, building on the complexity analysis in Section 5.5.

## Figures and Tables

**Table 1:** Wavelet scattering network properties.

Properties	Value
Signal length	15,001
Invariance scale	21 s
Sampling frequency	700 Hz
Quality factors	[8 1]
Optimize path	true

**Table 2:** Inference rule base for IT2FLC.

Error	dError				
	**dBN**	**dMN**	**dS**	**dMP**	**dBP**
BN	$U_{1}$	$U_{2}$	$U_{1}$	$U_{4}$	$U_{5}$
MN	$U_{1}$	$U_{2}$	$U_{5}$	$U_{4}$	$U_{5}$
S	$U_{3}$	$U_{3}$	$U_{2}$	$U_{4}$	$U_{4}$
MP	$U_{4}$	$U_{4}$	$U_{4}$	$U_{4}$	$U_{4}$
BP	$U_{5}$	$U_{5}$	$U_{5}$	$U_{5}$	$U_{5}$

**Table 3:** Execution times of optimization algorithms.

Algorithm	Average Execution Time (s)
GA	0.41
GD	0.26
Hybrid (GA + GD)	0.52

**Table 4:** Performance indices for the irreversible level.

Metrics	X Axis	Y Axis	Z Axis	Average
ISE	10.76	7.89	3.60	7.41
ITAE	84.36	75.58	49.95	69.97

**Table 5:** Classification criteria for degradation levels.

Level	ISE/ITAE
Normal	0
Minor Fault	0<x≤0.3
Moderate Fault	0.3<x≤0.6
Critical Fault	0.6<x<1
Irreversible Fault	1

**Table 6:** Performance indices for the actuator fault.

Metrics	X Axis	Y Axis	Z Axis	Average	Normalized Average
ISE	0.05	1.06	0.35	0.49	0.07
ITAE	2.04	18.49	17.17	12.57	0.18

**Table 7:** Performance indices for the sensor fault.

Metrics	X Axis	Y Axis	Z Axis	Average	Normalized Average
ISE	7.22	1.43	0.76	3.14	0.42
ITAE	62.71	21.88	20.29	34.96	0.50

**Table 8:** Performance indices for the multiple fault.

Metrics	X Axis	Y Axis	Z Axis	Average	Normalized Average
ISE	10.55	7.94	3.27	7.25	0.98
ITAE	83.95	72.82	48.88	68.55	0.98

**Table 9:** Faults classification from different feature extraction sources.

	Training				Test			
**Feature Extraction**	**BT**	**LD**	**MNN**	**CKNN**	**BT**	**LD**	**MNN**	**CKNN**
WSN + PCA	80.4%	80.4%	82.3%	77.0%	66.7%	78.0%	72.0%	63.1%
WSN + PCA + LDA	91.7%	91.1%	90.5 %	90.7%	91.1%	98.2%	95.2 %	97.0%
WSN + PCA + LDA + ML	100%	100%	100%	100%	100%	100%	100%	100%

**Table 10:** Characteristics of the classification algorithms.

	BT	LD	MNN	CKNN
Training Time (s)	3.82	1.83	3.09	0.95
Algorithm Size (kB)	166	5	8	29

**Table 11:** Diagnostic processing times.

	Preprocessing	Classifiers				Complete Diagnostic
	**WSN + PCA + LDA + ML**	**BT**	**LD**	**MNN**	**CKNN**	**(Preprocessing + Classification)**
Processing Time (ms)	54	39				93
			14			68
				34		88
					17	71

**Table 12:** Scaling factors reconfigured by the active fault-tolerant control.

Scaling Factor	Original Value	Actuator Fault	Sensor Fault	Multiple Fault
K*_e_*	1.3	1.25	1.20	1.26
K*_d_*	0.3	0.15	0.20	0.39
K*_i_*	1.5	1.42	1.30	1.65
K_*i*1_	1.4	1.51	1.21	1.38

**Table 13:** Performance indexes for the actuator fault reconfiguration.

Metrics	X Axis	Y Axis	Z Axis	Average	Normalized Average
ISE	0.03	1.03	0.25	0.44	0.06
ITAE	1.23	16.52	9.05	8.94	0.13

**Table 14:** Performance indexes for the sensor fault reconfiguration.

Metrics	X Axis	Y Axis	Z Axis	Average	Normalized Average
ISE	6.81	0.70	0.59	2.70	0.36
ITAE	62.63	16.20	18.87	32.57	0.47

**Table 15:** Performance indexes for the multiple fault reconfiguration.

Metrics	X Axis	Y Axis	Z Axis	Average	Normalized Average
ISE	9.56	0.48	0.47	3.50	0.47
ITAE	80.29	13.39	17.64	37.11	0.53

**Table 16:** Scaling factors obtained from optimization using GAs.

Parameter	Actuator Fault	Sensor Fault	Multiple Fault
K*_e_*	1.26	1.22	1.27
K*_d_*	0.16	0.22	0.43
K_*i*_	1.42	1.35	1.68
K_*i*1_	1.53	1.24	1.40

**Table 17:** Performance indexes obtained from optimization using GAs.

Scenario	ISE Average	ISE Average Normalized	ITAE Average	ITAE Average Normalized
Actuator fault	0.47	0.06	9.48	0.14
Sensor fault	2.79	0.38	32.93	0.47
Multiple fault	4.61	0.62	45.98	0.66

**Table 18:** Scaling factors obtained from optimization using GD.

Parameter	Actuator Fault	Sensor Fault	Multiple Fault
K_*e*_	1.29	1.17	1.30
K_*d*_	0.23	0.28	0.35
K_*i*_	1.40	1.42	1.55
K_*i*1_	1.44	1.36	1.36

**Table 19:** Performance indexes obtained from optimization using GD.

Scenario	ISE Average	ISE Average Normalized	ITAE Average	ITAE Average Normalized
Actuator fault	0.49	0.07	12.32	0.18
Sensor fault	3.01	0.41	33.81	0.48
Multiple fault	6.56	0.89	62.89	0.90

**Table 20:** Lyapunov exponents for different scenarios.

Scenario	Lyapunov Exponent
Initial conditions perturbation	−0.5053
Multiple fault	−0.1856
Multiple fault after control reconfiguration	−0.2776

**Table 21:** Actuator fault. Comparison of ISE index for fault-tolerant control between Sliding Mode and the proposed hybrid optimization.

ISE	Sliding Mode	Hybrid Optimization
X axis	3.67	0.03
Y axis	0.58	1.03
Z axis	0.18	0.25
Average	1.47	0.44

**Table 22:** Sensor fault. Comparison of ISE index for fault-tolerant control between Sliding Mode and the proposed hybrid optimization.

ISE	Sliding Mode	Hybrid Optimization
X axis	2.42	6.81
Y axis	7.86	0.70
Z axis	1.83	0.59
Average	4.04	2.70

**Table 23:** Multiple fault. Comparison of ISE index for fault-tolerant control between Sliding Mode and the proposed hybrid optimization.

ISE	Sliding Mode	Hybrid Optimization
X axis	10.01	9.56
Y axis	15.31	0.48
Z axis	4.46	0.47
Average	9.93	3.50

## Data Availability

The data presented in this study are contained in the article itself.
